# Apelin promotes diabetic nephropathy by inducing podocyte dysfunction *via* inhibiting proteasome activities

**DOI:** 10.1111/jcmm.12619

**Published:** 2015-06-23

**Authors:** Caixia Guo, Yu Liu, Wenjie Zhao, Shengnan Wei, Xiaoli Zhang, Wenying Wang, Xiangjun Zeng

**Affiliations:** a Department of Pathophysiology and Pathology, Capital Medical UniversityBeijing, China; b Department of Cardiology, Beijing Tiantan Hospital, Capital Medical UniversityBeijing, China; c Department of Urology, Beijing Friendship Hospital, Capital Medical UniversityBeijing, China

**Keywords:** diabetic nephropathy, podocyte, proteasome, ER stress, apelin, APLNR

## Abstract

Podocyte injuries are associated with progression of diabetic nephropathy (DN). Apelin, an adipocyte-derived peptide, has been reported to be a promoting factor for DN. In this study, we aim to determine whether apelin promotes progression of DN by inducing podocyte dysfunction. kk-Ay mice were used as models for DN. Apelin and its antagonist, F13A were intraperitoneally administered for 4 weeks, respectively. Renal function and foot process proteins were analysed to evaluate the effects of apelin on kk-Ay mice and podocytes. Apelin increased albuminuria and decreased podocyte foot process proteins expression in kk-Ay mice, which is consistent with the results that apelin receptor (APLNR) levels increased in glomeruli of patients or mice with DN. In cultured podocytes, high glucose increased APLNR expression and apelin administration was associated with increased permeability and decreased foot process proteins levels. All these dysfunctions were associated with decreased 26S proteasome activities and increased polyubiquitinated proteins in both kk-Ay mice and cultured podocytes, as demonstrated by 26S proteasome activation with cyclic adenosine monophosphate (cAMP) or oleuropein. These effects seemed to be related to endoplasmic reticulum (ER) stress, as apelin increased C/EBP homologous protein (CHOP) and peiFα levels while cAMP or oleuropein reduced it in high glucose and apelin treated podocytes. These results suggest that apelin induces podocyte dysfunction in DN through ER stress which was induced by decreased proteasome activities in podocytes.

## Introduction

Diabetic nephropathy (DN) is one of the major complications of diabetes mellitus and is the most common cause of end-stage renal diseases 1. Central to DN progression is glomerular injury 2, which includes injuries of endothelial cells, tubulointerstitial cells, podocytes and mesangial cells, while podocyte injuries are critical in glomerular dysfunction in DN. However, it is disputed that how podocyte is injured in diabetes 3.

Various pathogenic factors have been reported to account for podocyte injuries in DN 4–7. However, few studies have ever evaluated the actions of adipose tissue on podocyte injuries in DN. The peptide apelin, described as an adipokine due to the production and secretion by isolated mature adipocytes 8, has been reported to be positively related to urinary albumin creatinine ratio (ACR) in diabetic patients 9,10. Our previous works also found that apelin displayed promoting effects on DN by enhancing the permeability of glomerular endothelial cells 10. As emerging evidence suggest that endothelial cell and podocyte injuries in DN are closely related to each other 11, we hypothesized that apelin might promote the podocyte dysfunction in DN as well.

Podocyte injuries consists of apoptosis, detachment, hypertrophy, effacement and loss of foot process proteins 2,3, of which the intracellular protein regeneration and degradation are the main alternations 12,13. The inability of podocytes to proply fold, modify assemble native proteins lead to accumulation of misfolded protein in endoplasmic reticulum (ER) 14. Under these conditions of ER stress, the ER attempts to restore disrupted homeostasis by inhibiting translation and increasing protein degradation. Multiple systems exist for proteolysis, the best described of which is the highly conserved non-lysosomal proteolytic ubiquitin-proteasome system (UPS) which degrades ubiquitin-conjugated proteins by proteaomes 15. Studies have revealed that podocyte injuries were associated with accumulation of monoubiquitinated and polyubiquitinated proteins which resulted from impaired proteasome activities 7,16. The proteasome activities could be activated by cAMP 17 and apelin/APLNR has been reported to mainly act through Gαi and decrease cAMP 18. Therefore, apelin might promote podocyte dysfunction through impairing proteasome activities. The goal of this study was to testify apelin induces podocyte injuries in DN by inhibiting proteasome activities which would result in ER stress.

## Materials and methods

### Human subjects

Subjects were recruited from inpatients that were operated on kidney because of cancer. The participants have to meet diagnosis criteria of chronic kidney diseases recommended by the Kidney Disease Outcome Quality Initiative 19, simply defined as urine albumin>300 μg/mg with history of diabetes for at least 1 year. Clinical healthy individuals were negative to urine albumin and no history of known diabetes or kidney disease (serum creatinine, ≤1.4 mg/dl). Exclusion criteria included cardiovascular diseases, cerebrovascular diseases, hypertension, metabolic diseases and inflammatory diseases. We enrolled 20 patients with type 2 diabetes and 20 patients without type 2 diabetes (the detailed information was in Table1). The study protocol was approved by the ethics committee of Capital Medical University, and informed consent was given by each patient before enrollment. The consent was verbal because the samples were the residues of clinical test specimens. Thus, no extra treatment to the patients was required. The patients signed a sheet that presented the selected cases in table format. The institutional review board of Capital Medical University waived the need for written informed consent from the participants.

**Table 1:** The index of enrolled patients with or without type 2 diabetes

	Patients with type 2 diabetes		Patients without type 2 diabetes	
	Male (*n* = 11)	Female(*n* = 9)	Male (*n* = 10)	Female (*n* = 10)
Age	57.5 ± 6.6	55.6 ± 9.6	55.1 ± 8.0	55.7 ± 9.3
Body mass index (BMI) (kg/m^2^)	29.8 ± 2.04	29.7 ± 5.9	29.6 ± 3.1	26.4 ± 8.4
Systolic blood pressure (mmHg)	135 ± 8.0	130 ± 10	130 ± 8.9	130 ± 11
Diastolic blood pressure (mmHg)	80 ± 7.0	80 ± 11.5	75 ± 10.5	80 ± 11.3
Triglyceride	1.20 ± .023	1.30 ± 0.31	1.15 ± 0.26	0.94 ± 0.33
Cholestrol	4.20 ± 0.61	4.32 ± 0.56	4.21 ± 0.57	3.57 ± 0.61
Low density lipoprotein	2.60 ± 0.14	2.45 ± 0.21	2.53 ± 0.21	2.60 ± 0.22
High density of lipoprotein	1.13 ± 0.11	1.25 ± 0.13	1.22 ± 0.14	1.26 ± 0.15
Fasting blood glucose	6.61 ± 0.56	7.35 ± 0.53	5.35 ± 0.42	5.15 ± 0.23
Glycosylated haemoglobin	7.65 ± .68	7.39 ± 0.54	5.11 ± 0.35	4.89 ± 0.44
Albumin creatinine ratio (μg/mg creatinine)	485 ± 21.5	512 ± 55.3	15.9 ± 0.98	15.7 ± 0.87

### Experimental animals

All animal studies followed the Animal Care and Use Committee of Capital Medical University (20100610). All animals received humane care, and the experimental protocol was approved by the Committee of Laboratory Animals according to institutional guidelines.

The kk-Ay mouse is considered to be a polygenic model for human type 2 diabetes model 20. It exhibits marked obesity, glucose intolerance, severe insulin resistance, dyslipidaemia and hypertension. Kk-Ay mouse also develops renal disease characterized by moderate albuminuria with mild glomerular pathology and podocyte loss 20,21.

Male kk-Ay mice and control C57BL/6J at 12 weeks of age mice were purchased from Capital Medical University (Beijing, China). Mice were fed on semi-purified moderately high-fat diet containing 24% kcal fat and 0.2% cholesterol. Mice were randomized according to albumin/creatinine at 12 weeks of age and were killed at 16 weeks of age.

C57BL/6J mice were classified as normal control (C57BL), and kk-Ay mice were considered DN when their urine ACR was ≥300 μg/mg. kk-Ay mice were then randomly divided into DN control group (kk group, *n* = 10), which were intraperitoneally injected with vehicle, apelin treatment group (kk+apelin), which were intraperitoneally injected with apelin-13 (A6469; Sigma-Aldrich, St. Louis, MO, USA, 30 μg/kg/day) for 4 weeks and F13A treatment group (kk+F13A), which were intraperitoneally injected with F13A (the antagonist of apelin-13,057-29; Phoenix Pharmaceuticals, Strasbourg France, 25 μg/kg/day) for 4 weeks.

### Biochemical characterization

The level of fasting serum creatinine and fasting bodyweight were measured at the end of the experiments. The urine samples were collected for a period of 24 hrs using a mouse metabolic cage (CLEA Tokyo, Japan). Urinary albumin, and creatinine were measured by immunoassay (DCA 2000 system; Siemens AG, Munich, Germany). All analyses were performed in accordance with the manuals provided by the manufacturers. The urinary ACR = urinary albumin (μg)/urinary creatinine (mg). Ccr (Creatinine clearance ratio, ml/min./kg) = [urinary Creatinine (mg/dl) × urinary volume (ml)/serum Creatinine (mg/dl)] × [1000/bodyweight (g)] × [1/1440 (min.)].

### Pathological examination

Kidneys were excised carefully without any damage and stored in 10% neutral buffered formalin after being washed with PBS. Sections of 5 μm were cut and stained with periodic acid Schiff (PAS) base for pathological observations. An additional aliquot of normal kidney was frozen in optimum cutting temperature compound (OCT) for immunofluorescent staining. A separate aliquot of kidney cortical tissue was cut into 1-mm^3^ pieces and fixed in 2.5% glutaraldehyde in Millonig solution and embedded in PolyBed 812 (Polysciences Inc., Taipei, Taiwan) for EM (electronic microscope) analysis.

Glomeruli from 16-week-old male mice from every group were screened on PAS staining. Each animal was evaluated for the presence of glomerular deposits. Deposits were evaluated by giving a grade for the presence of deposits on a scale of one to four: 1 = 25% of the glomerulus; 2 = 25–50%; 3 = 51–75%; 4 = 75% of the glomerulus contains deposits. A total of 50 glomeruli per section were evaluated.

### Podocyte cell culture

Conditionally immortalized mouse podocytes, kindly provided by P. Mundel (Mt. Sinai School of Medicine, New York, USA), were cultured as previously described 22. When podocytes were well-differentiated, they were serum-starved overnight with serum-free DMEM with NG (5.5 mmol/l D-glucose) or high glucose (HG: 25 mmol/l D-glucose) for 24 hrs. Cells were then modulated with apelin (1.0 nmol/l) or F13A (1.0 nmol/l) and 50 μmol/l 8-Br-cAMP (B7880; Sigma-Aldrich) or oleuropein (0.5 μg/ml, 32619-42-4; Yuanmu Co. Ltd., Shanghai, China) for 48 hrs and harvested for the following assays.

### Permeability of podocytes

A modification of a previously published protocol was adopted 23. Differentiated podocytes were plated on type I collagen coated 24-well transwell plates (P18P01250; Millipore, Carrigtwohill, Ireland). The upper compartment was refilled with 0.25 ml RPMI medium1640 supplemented with 0.5 mg/ml FITC-BSA (FITC labeled bovine serum albumin, A9771; Sigma-Aldrich). The lower compartment was filled with 0.5 ml RPMI medium 1640 containing 0.5 mg/ml unlabelled BSA and incubated for 3 hrs at 37°C. FITC-BSA concentration in the lower compartment was determined using a Bio-Tek protein assay (Bio-Tek Laboratories, Hongkong, China) at 492/520 nm absorption/emission wavelengths.

### Proteasome activities

The activities of chymotrypsin-like, caspase-like and trypsin-like peptidases of the proteasome were assayed according to modified published protocols 24. 20 μg protein homogenate was mixed with 25 mmol/l Tris-HCl, pH 7.0, and 40 mmol/l proteasome peptidase substrates, up to a total volume of 100 μl, which was then incubated for 1 hr at 37°C. Then, the fluorescence of each solution was monitored by assessing the release of aminoluciferin (AMC) using spectrophotometry (HitachF-2000; Hitachi Instruments, Tokyo, Japan) at an excitation wavelength of 380 nm and an emission wavelength of 440 nm. All results were standardized to the fluorescence intensity by an equal volume of free AMC (SCP0225; Sigma-Aldrich).

### Immunohistochemistry

Tissue sections at 5 μm were used to perform immunostaining for APLNR and synapotopodin with rabbit anti APLNR (sc-33823; Santa Cruz Biotechnology, Santa Cruz, CA, USA) and rabbit anti-synapotopodin (sc-50459; Santa Cruz Biotechnology). Second antibodies were donkey anti-rabbit-TR and donkey anti rabbit-HRP. Images were obtained using a microscope (Olympus BX-63, Tokyo, Japan).

### Western blotting of protein expression

The proteins from tissues or cells were fractionated by electrophoresis on 10% SDS-PAGE and electro-blotted to polyvinylidene difluoride filter membranes and incubated with the primary antibody at 4°C, and then with a HRP-conjugated secondary antibody. Primary antibodies: rabbit anti-APLNR, goat anti-nephrin (sc-32529; Santa Cruz Biotechnology), rabbit anti-synapodocin (sc-50459; Santa Cruz Biotechnology), rabbit anti-podocin (sc-21009; Santa Cruz Biotechnology), rabbit anti-zonula occludens-1 (ZO-1) (sc-8146; Santa Cruz Biotechnology), rabbit anti-Wilm’s tumor protein (WT1) (sc-192; Santa Cruz Biotechnology), mouse anti-Ub (MAB1510; Millipore), rabbit anti-k48-linkage specific polyubiqutinated protein (k48, 8081; Cell Signaling Technology, Danvers, MA, USA), mouse anti-CHOP (2895; Cell Signaling Technology) and rabbit anti-phospho-eIF2α (3398; Cell Signaling Technology). Densitometry was performed with Image J software (NIH, USA). To verify equal loading, antibody to GAPDH was used.

### Statistics

Data were summarized as mean ± SD. A *p*-value less than 0.05 was considered significant. All reported *p*-values are two-sided. Analyses were carried out using SPSS version 13.0 for the PC (Armonk, NY, USA). Differences between different groups were evaluated for significance using independent *t*-test or one-way anova and Newman–Keuls post-hoc tests.

## Results

### The expression and location of APLNR in renal tissue

**Figure 1:**
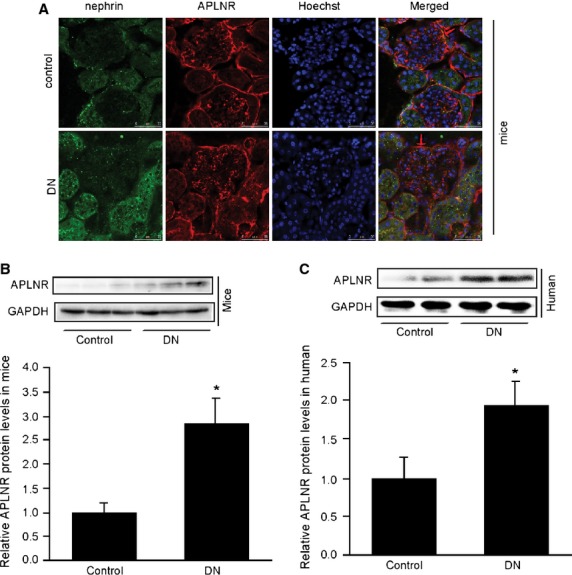
Increased APLNR level in mice and human kidney. (A) Immunostating of APLNR in mice kidney. (B) The APLNR levels in the kidney of type 2 diabetic mice (kk-Ay mice) was significantly increased by 2.7-fold compared with that of control mice (C57BL). The data are expressed as the means ± SD (*n* = 6, *p < 0.01 *versus* control group). (C) APLNR level in the kidney of type 2 diabetic patients was significantly increased by 1.9-fold compared with that of control patients. The data are expressed as the means ± SD (*n* = 6, *p < 0.01 *versus* control group).

Prior to exploring the promoting effects of apelin in DN, we performed immuno-staining and Western blotting to examine the protein levels of APLNR in kidneys of diabetic human and mice. Immuno-staining detected increased APLNR level in podocytes of type 2 diabetic mice kidney (Fig.1A). APLNR expression was confirmed by Western blotting analysis in kidneys of mice and patients with DN. APLNR expression was increased in kidney of mice with DN (Fig.1B) and patients with DN (Fig.1C).

### Apelin replacement aggravated ACR and Ccr

**Figure 2:**
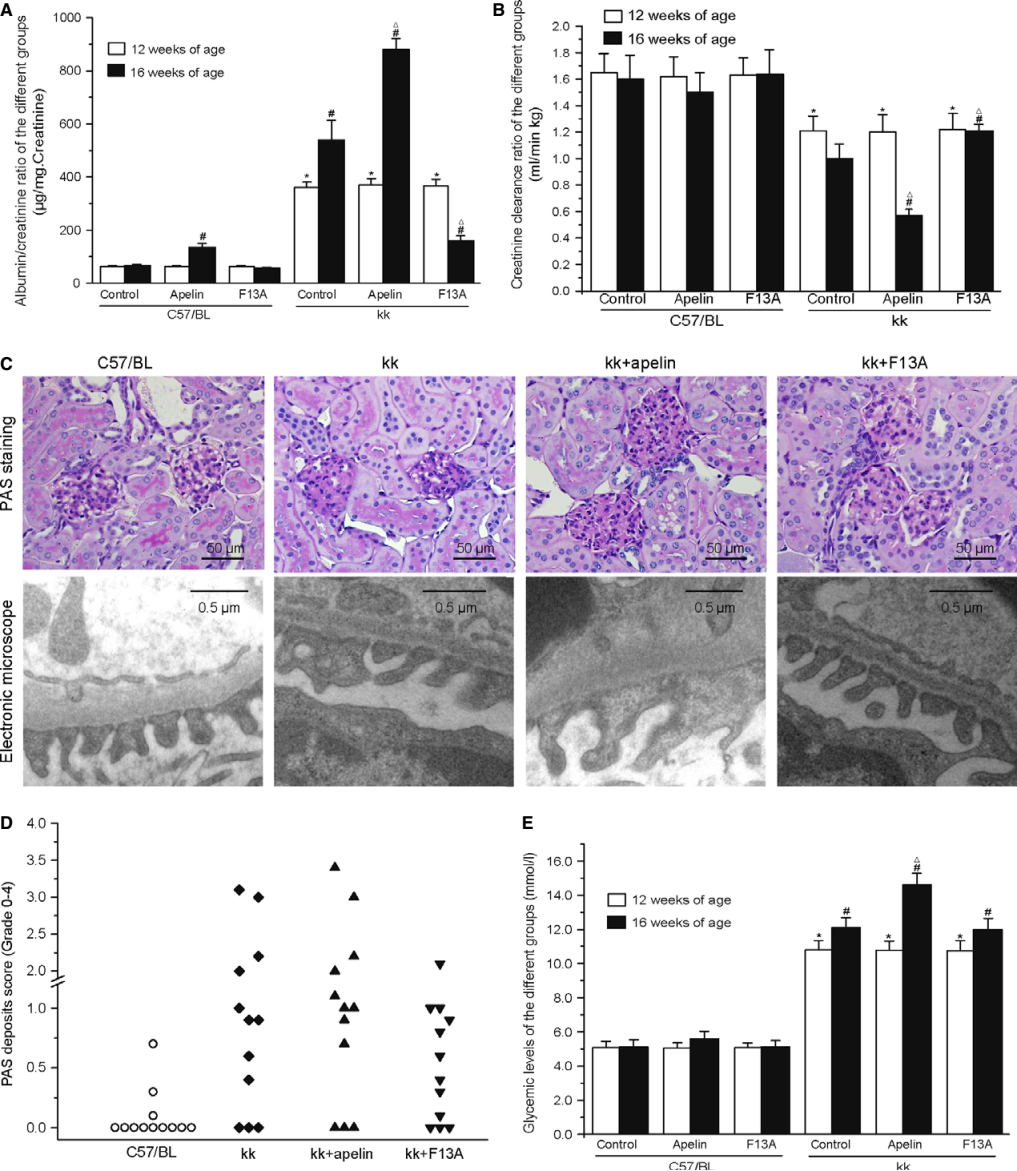
Apelin aggravated renal dysfunction. (A) Apelin significantly increased the albuminuria both in kk-Ay and control mice while F13A reversed it in kk-Ay mice (*n* = 10). (B) Apelin decreased Ccr in kk-Ay mice while F13A reveresed it (*n* = 10). (C) Representative photographs of kidney section from different groups. Apelin significantly increased the thickening of the GBM and podocyte effacement in kk-Ay mice while F13A reversed it. (D) Scoring of the severity of the deposits in PAS staining of mice in every group. The score represents the mean percentage of the total of 50 glomeruli affected (grade 0–4; *n* = 12). (E) Glycaemic levels of the different groups of mice before and after apelin or F13A treatment (*n* = 12). *p < 0.01 *versus* C57BL mice of 12 weeks old, ^#^*p* < 0.01 *versus* C57BL mice of 16 weeks old, ^Δ^*p* < 0.01 *versus* kk-Ay mice of 16 weeks old.

We examined whether apelin aggravated DN or promoted renal dysfunction independently. At the age of 12 weeks old, C57BL and kk-Ay mice showed different ACR (62.3 ± 0.3 μg/mg and 365.3 ± 2.9 μg/mg) and Ccr (1.6 ± 0.01 ml/min./kg and 1.2 ± 0.01 ml/min./kg) with statistical significance and without any significance between C57BL and kk-Ay mice in dfferent goups. After apelin treatment, the ACR was increased from 540 ± 74 μg/mg to 880 ± 41 μg/mg in kk-Ay mice and from 67 ± 4 μg/mg to 136 ± 14 μg/mg in C57BL mice (Fig.2A). Apelin decreased Ccr from 1.0 ± 0.11 ml/min./kg to 0.57 ± 0.047 ml/min./kg in kk-Ay mice and from 1.60 ± 0.18 ml/min./kg to 1.50 ± 0.15 ml/min./kg in C57BL mice (Fig.2B). F13A decreased the ACR to 160 ± 18 μg/mg in kk-Ay mice and 56 ± 3 μg/mg in C57BL mice (Fig.2A). F13A increased Ccr to 1.21 ± 0.047 ml/min./kg in kk-Ay mice and 1.64 ± 0.18 ml/min./kg in C57BL mice (Fig.2B). These results indicate that increased apelin/APLNR expression might contribute to renal dysfunction independently and aggravate the progression of DN.

### The effects of apelin on GBM and podocyte injury

The glomerular pathological changes in DN were characterized by glomerular basement membrane (GBM) thickening and inflammatory cell infiltration. GBM are mainly composed of glycoprotein, collagen and elastin. As shown in Figure2C, The glomeruli are enlarged, with thickened GBM, and show increased cell numbers and infiltrating leukocytes such as mononuclear cells and lymphocytes in kk-Ay mice. Treatment with apelin aggravated the pathological changes while F13A attenuated them. These results indicate that apelin might aggravate the renal dysfunction in kk-Ay mice by exacerbating the GBM thickening.

The severity of the deposits varied among the groups: C57BL control group (mean: 0.09 ± SE 0.06), kk-Ay mice group (1.18 ± 0.33), kk+apelin group (1.28 ± 0.33) and kk+F13A group (0.6 ± 0.18), and the severity between mice within one group varied as well (Fig.2D). The C57BL and kk-Ay mice were also evaluated at 12 weeks old to study the progression of GBM thickening, showing as C57BL (0.07 ± 0.07) and kk-Ay mice (0.96 ± 0.32), depicting the influence of apelin on GBM thickening.

**Figure 3:**
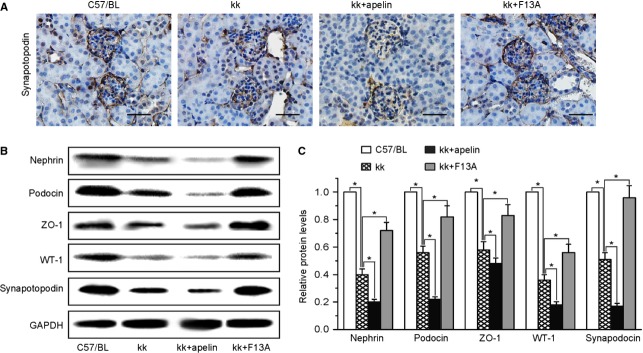
The effects of apelin on foot process proteins in kk-Ay mice. (A) Representative images of immunostaining for synapotopodin in kidneys. (B) Representative images of western blotting for foot process proteins. (C) Graphs show densitometry of the ratio of nephrin, podocin, ZO-1, WT-1 and synapotopodin to GAPDH in mouse kidney (mean ± SD, *n* = 3, *p < 0.05).

As podocytes are important for glomerular filtration, we examined foot process with electronic microscope. Foot process effacement and fusion were aggravated in kk-Ay mice with apelin treatment and prevented with F13A treatment (Fig.2C). The expression level of foot process proteins including nephrin, podocin, ZO-1, WT-1 and synapotopodin in mice were significantly decreased in kk-Ay mice (to 40%, 59%, 63%, 37% and 51% of C57BL mice, Fig.3), and apelin treatment further decreased the expression levels of these proteins (to 21%, 23%, 49, 19% and 17% of C57BL mice, Fig.3) and F13A restored the expression of these proteins (to 73%, 81%, 84%, 58% and 95% of C57BL mice, Fig.3). These results suggest that apelin levels might be one of the causes of podocyte dysfunction in DN.

### Direct effects of apelin on podocytes

Podocytes play a major role in the glomerular filtration barrier 25 and podocyte injuries are a key element of DN 26–28. To confirm the APLNR expression in podocytes, we examined the APLNR expression in cultured podcytes. The expression level of APLNR was increased by high glucose treatment and not changed by apelin treatment in podocytes (Fig. S1). The direct effects of apelin on podocyte permeability and foot process proteins showed that apelin dose-dependently increased the BSA-FITC filtrated through podocytes (Fig. S2A) and decreased foot process proteins in podocytes (Fig. S2B and C). These data indicate a direct action of apelin on podocytes independent of the systemic and/or metabolic aspects.

**Figure 4:**
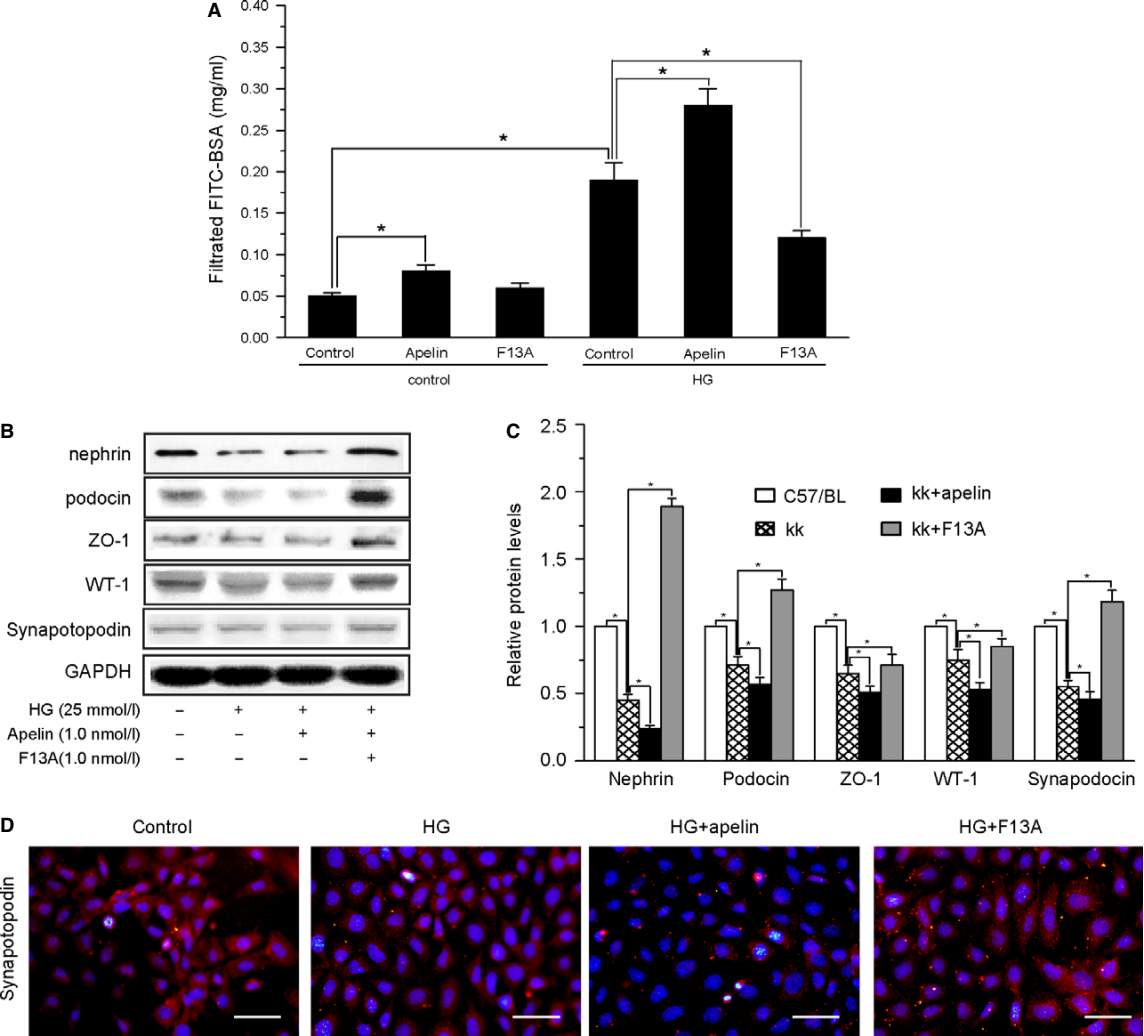
The effects of apelin on permeability and levels of foot process proteins in podocytes. (A) Albumin permeability was increased by HG and aggravated by apelin while reversed by F13A (*n* = 6). (B) Representative images of western blotting for foot process proteins in podocytes. (C) Graphs show densitometry of the ratio of nephrin, podocin, ZO-1, WT-1 and synapotopodin to GAPDH in podocytes (*n* = 3; mean ± SD, *p < 0.05).

Furthermore, podocyte permeability was significantly increased with high glucose (by 2.6-fold, Fig.4A) and apelin (by 4.2-fold, Fig.4A) treatment. F13A reversed the increasing permeability of podocyte (decreased by 39% compared with HG group, Fig.4A). These data indicate that apelin might aggravate the podocyte dysfunction in DN.

As foot process proteins are the important functional components of podocytes 29, we examined the expression levels of foot proteins in differentiated podocytes treated with HG and apelin or F13A. The results showed that HG decreased the five foot process proteins: nephrin, podocin, ZO-1, WT-1 and synapotopodin in cultured podocytes (to 48%, 68%, 61%, 75% and 54% of control cells, Fig.4B and C) and apelin aggravated the decreasing of these proteins (to 23%, 56%, 52%, 53% and 49% of control cells, Fig.4B and C) while F13A restored it (to 180%, 127%, 74%, 89% and 124% of control cells, Fig.4B and C). These results suggest that apelin induced the progression of podocyte dysfunction by decreasing the foot process proteins in podocytes.

### Apelin inhibited proteasome activities in kidney

**Figure 5:**
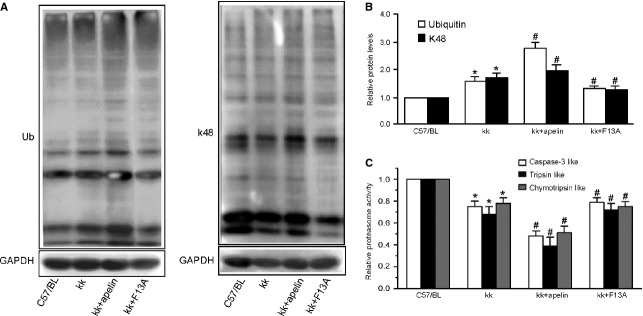
Apelin impairs proteasome peptidase activities in kk-Ay mice. (A) Polyubiquitinated proteins were increased in kk-Ay mice and aggravated by apelin while reversed by F13A (*n* = 3). (B) 26S proteasome activities are indicated as percentages of those in C57BL mice. All proteasome activities decreased in kk-Ay mice compared with C57BL mice and aggravated by apelin while reversed by F13A (*n* = 8; mean ± SD, *p < 0.05 *versus* control, ^#^*p* < 0.05 *versus* kk-Ay mice).

Because UPS impairment has been reported to be involved in podocyte dysfunction and albuminuria 7,16, we sought to determine whether apelin regulates proteasome activities in kk-Ay mice. Compared with control group, all of the proteasome activities were decreased by 24%, 34% and 22% (for caspase-like, trypsin-like and chymotrypsin-like activities, Fig.5B) in kk-Ay mice, as would be expected from the results showing an increased pool of ubiquitinated proteins (by 51% and 60% for ubiquitinated and k48 Linked proteins, Fig.5A and B). Furthermore, all of the proteasome activities were further decreased by 52%, 60% and 49% (for caspase like, tripsin like and chemotripsin like activities, Fig.5C) with apelin treatment and were restored with F13A treatment to 79%, 73% and 76% of control, which were confirmed by increase or decrease of polyubiquitinated proteins (Fig.5A and B). These results suggest that apelin might aggravate podocyte dysfunction in DN through inhibiting proteasome activities followed with aggregation of polyubiquitinated proteins.

### Apelin attenuated proteasome activities which was inhibited by HG in podocytes

**Figure 6:**
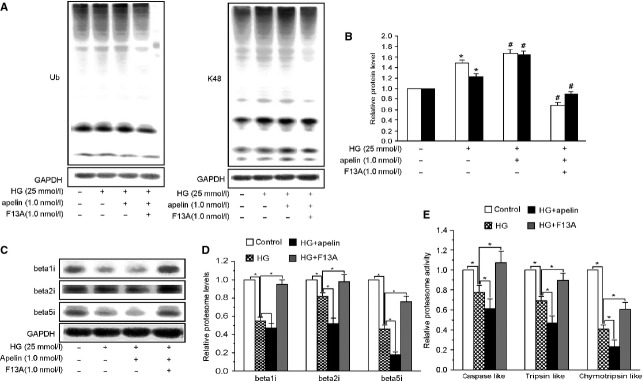
Apelin impairs proteasome peptidase activities in podocytes. (A) Polyubiquitinated proteins were increased in high glucose (HG:25mM D-glucose) treated podocytes and aggravated by apelin while reversed by F13A (*n* = 3). (B) 26S proteasome activities are indicated as percentages of those in normal glucose (NG: 5.5mM D-glucose). All proteasome activities decreased in HG treated podocytes compared with NG and aggravated by apelin while reversed by F13A (*n* = 6). (C) Immunosubunits of proteasome were decreased in HG treated podocytes and further decreased by apelin while reversed by F13A (*n* = 3; mean ± SD, *p < 0.05 versus control group, #*p* < 0.05 versus HG treated group).

To evaluate whether apelin directly regulated proteasome activities in podocytes, polyubiquitinated proteins and proteasome activities were assessed in podocytes. Apelin dose- dependently increased polyubiquitinated proteins (Fig. S3A and B) and decreased proteasome activities (Fig. S3C) in podocytes. Polyubiquitinated protein levels were increased by 48% and 21% of control (for ubiquitinated and K48-linked proteins) after HG treatment, and apelin treatment aggravated these increase by 64% and 62% of control (for ubiquitinated and K48-linked proteins) while F13A prevented it by 32% and 18% of control (for ubiquitinated and K48-linked proteins; Fig.6A and B). Correspondingly, proteasome activities were decreased to 78%, 69% and 40% of control (for caspase like, tripsin like and chemotripsin like activities) after HG treatment, and apelin aggravated these decrease to 61%, 48% and 23% of control (for caspase-like, tripsin-like and chemotripsin-like activities) while F13A restored it to 108%, 89% and 60% of control (Fig.6E). These results indicate that apelin impaired the proteasome activities in podocyte of DN.

Among the proteasome subunits, immunoproteasome subunits (β1i, β2i and β5i) are responsible for inducing proteasome activities 30, therefore, we detected the immunoproteasome subunits in cultured podocytes. The results showed that HG decreased the expression levels of β1i, β2i and β5i levels to 54%, 82% and 46% of control, and the reduction was aggravated by apelin to 47%, 52% and 19% of control and restored by F13A to 94%, 98% and 75% of control (Fig.6C). These results indicate that apelin inhibited the proteasome activities by decreasing the expression levels of immunoproteasome subunits in podocytes.

### Role of proteosome activities in podocyte injuries induced by apelin

**Figure 7:**
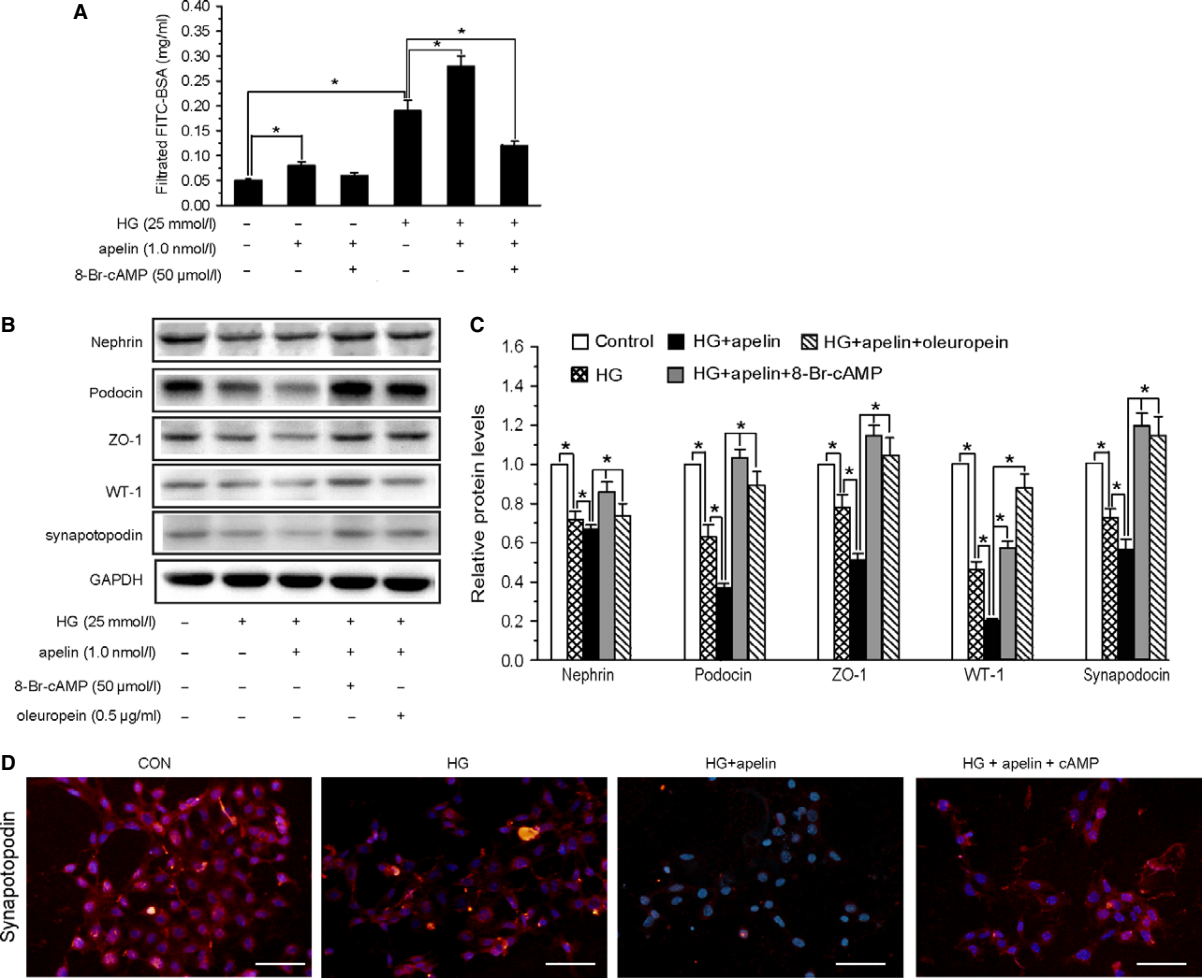
Apelin aggravated podocytes injuries through inhibiting proteasome activities. (A) Apelin increased the albumin permeability of podocytes treated with HG, which was inhibited by cAMP (*n* = 6). (B) Representative images of western blotting for foot process proteins in podocytes. (C) Graphs show densitometry of the ratio of nephrin, podocin, ZO-1, WT-1 and synapotopodin to GAPDH in podocytes (*n* = 3). (D) Representative images of immunostaining for synapotopodin in podocytes (mean ± SD, *p < 0.05).

It has been reported that cAMP stimulates the proteasome activities 31, and apelin act with APLNR to activate Gαi and decreases cAMP levels. cAMP was added to increase proteasome activities in podocytes. The results showed that HG and apelin induced increasing of polyubiquitinated protein and decreasing of proteasome activities were reversed by cAMP (Fig. S4). The increased permeability of podocytes induced by HG and apelin was reversed by cAMP (to 0.13 ± 0.02 mg/ml, Fig.7A) and the foot process proteins, nephrin, podocin, ZO-1, WT-1 and synapotopodin, were reversed back (to 89%, 107%, 113% and 120% of normal, Fig.7B–D). These results suggest that apelin induced podocyte dysfunction in DN through inhibiting cAMP.

Besides activating proteasomes, cAMP possesses much more potential functions in cells 32–34. To prove that inhibiting proteasome activities is one of the key pathways induced by apelin, which will lead to podocyte injures in DN, we used oleuropein to activate proteasome activities 35,36 to find whether the podocyte injures induced by apelin were reversed or not. The results showed that oleuropein restored the foot process proteins, nephrin, podocin, ZO-1, WT-1 and synapotopodin, decreased by HG and apelin (to 72%, 91%, 104%, 90% and 115% of normal, Fig.7B–D). These results indicate that apelin aggravate podocyte injuries in diabetes by inhibiting proteasome activities.

### ER stress was induced by decreased proteosome activities in podocytes

**Figure 8:**
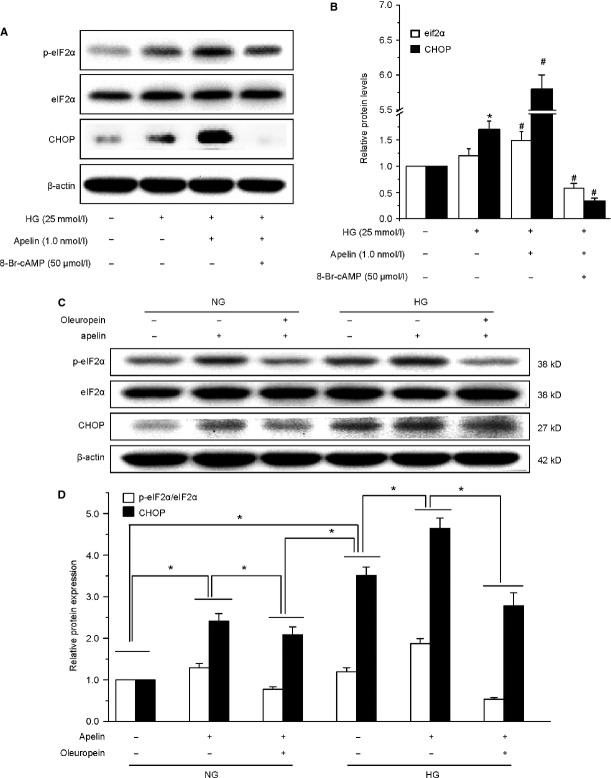
Apelin stimulated endoplasmic reticulum stress in podocytes, which was inhibited by cAMP or oleuropein. (A and C) Representative images of western blotting for eif2α and CHOP. (B and D) Graphs show densitometry of the ratio of eIF2α and CHOP to GAPDH in podocytes (mean ± SD, *n* = 3, *p < 0.05 versus control group, #*p* < 0.05 versus HG treated group).

Among the pathways implicated in UPS action, ER stress appears to have a major role for kidney dysfunction 16. The baseline ER stress markers, phospho-eIF2α and CHOP, in differentiated podocytes were increased by high glucose and were further increased by addition of apelin, but the increase was prevented by cAMP or oleuropein (Fig.8). These results suggested that apelin aggravated podocyte injuries by inducing ER stress which was caused by decreased proteasome activities.

## Discussion

We had reported that apelin concentration in serum and kidney was increased in DN 10. In the present study, we reported that APLNR level was increased within the glomeruli in patients and mice with DN, and apelin aggravated ACR, Ccr and GBM thickning in kk-Ay mice,from which we concluded that excessive apelin/APLNR contributes to renal dysfunction in DN. As apelin is an adipokine and could be increased with body mass 8, therefore, these effects might be present in obese chronic kidney disease too. However, it is controversial that apelin/APLNR induce or prevent the progression of DN 9,10,37. As podocyte dysfunction is critical in renal dysfunction of DN 29, we aimed to verify the effects of apelin/APLNR on podocyte dysfunction in DN.

First of all, we detected the effects of apelin on glomerular filtration. The results found that microalbuminuria and podocyte foot process effacement were aggravated *via* treatment with apelin and abrogated *via* treatment with F13A as shown in Figure2C and D. Furthermore, foot process proteins were further down-regulated by apelin and extenuated by F13A in kk-Ay mice as shown in Figure3. These effects were confirmed by cultured podocytes, which showed that apelin decreased the expression of foot process proteins in podocytes and increased the FITC-BSA filtrating through monolayered podocytes as shown in Figure4. These results suggest that apelin/APLNR does contribute to glomerular filtration membrane injuries through aggravating podocyte injuries in DN.

Then, the problem is how apelin/APLNR induces the podocyte injuries. Apelin/APLNR is reported to inhibit cAMP by activating Gαi 38, and cAMP has been reported to increase the UPS activities 31. The UPS selectively degrades damaged or abnormal proteins 39, and sometimes the proteasome might be impaired or ‘choked’ by certain factors 16 which will induce the ER stress. And the ER stress has been reported to be responsible for podocyte injuries 40,41. Therefore, we assume that apelin/APLNR might impair the proteasome by inhibiting cAMP and induce the ER stress in podocyte to aggravate the progression of DN.

Our results showed that apelin increased the polyubiquitinated proteins in kk-Ay mice while F13A restored it. At the same time, an even more reduction in proteasomal enzymatic activities in kk-Ay mice was observed after apelin treatment (as shown in Fig.5). These results suggest that impairing proteasome activities may contribute to the apelin-aggravated podocyte injuries in DN. Meanwhile, we also observed that apelin increased polyubiquitinated proteins and decreased proteasome activities in cultured podocytes (as shown in Fig.6A, B and E). These results imply that decreased proteasome activities in podocytes, which are associated with increased ubiquitinated proteins content, might be the key pathway involved in apelin induced podocyte injury in DN. These results are consistent with the podocyte injury in rat membrane nephropathy 7.

To validate how apelin decreases the proteasome activities in podocytes, we detected the expression of immunoproteasome subunits (β1i, β2i and β5i) in podocytes. The results showed that apelin further reduced β1i, β2i and β5i levels decreased by HG (as shown in Fig.6C and D). These results suggest that apelin inhibits the proteasome activities by decreasing the levels of immunoproteasome subunits in podocytes.

Then, what intermediates apelin/APLNR reduced proteasome activities? cAMP has been reported to stimulate the proteasome activities 31, and apelin acts with APLNR to activate Gαi and decrease cAMP levels. Therefore, we assumed cAMP as the mediation for aplein/APLNR and proteasome activities. The results showed that cAMP reversed the increased BSA-FITC filtration and decreased foot process proteins in apelin treated podocytes (as shown in Fig.7). Thus, we conclude that decreased cAMP levels in podocytes by apelin mediates the inhibition of proteasome activities and results in podocyte dysfunction.

The remaining problem is how the dysfunction of proteasome leads to podocyte injury. Recent studies imply that complement-induced podocyte injury is associated with protein misfolding in the ER, and the enhancement of ER chaperoning capacity reduces injuries 42,43. Under ER stress, variety of proteins were up-regulated or phosphorated to restore ER homestasis 14,44. We detected CHOP and phosphorate of eIF2α, and the results indicated that CHOP and p-eIF2α levels were increased by apelin in high glucose treated podocytes (as shown in Fig.8A). These effects were reversed by activation of protesome activities with cAMP (as shown in Fig.8B). These findings confirmed us that apelin induced podocyte dysfunctions in DN might be due to the apelin triggered ER stress. There are other ER stress proteins 45,46, such as GRP78, GRP94 and ORP150, which were reported to be involved in podoctye injury. Whether the other ER stress proteins were involved in apelin/APJ induced podocyte injury needs more further studies.

However, there are many other effects that could be induced by cAMP besides stimulating proteasome activities 32–34. To verify that proteasome is the downstream pathway in apelin treated podocytes, we used oleuropein to activate proteasome and found that both foot process proteins and ER stress markers were reversed just like the effects of cAMP (as shown in Figs7B, C and 8C, D). These results suggest that apelin/APLNR decrease proteasome activities by inhibiting cAMP.

In conclusion, apelin/APLNR contributes to albuminuria in DN. Apelin plays a promoting role to enhance albuminuria by directly affecting podocyte function *via* inhibiting the proteasome activities which subsequently induced ER stress. Our results provide a pathological evidence for the apelin-protesome-ER stress pathway to promote albuminuria and potentially affect early DN. Therefore, apelin/APLNR might be a potential target for preventing the progressin of DN.

## Supporting Information

**Figure S1** (A) The immunohistochemical assay of APLNR expression in podocytes.

**Figure S2** Effects of apelin on podocyte permeability and foot process proteins expression in podocytes.

**Figure S3** Apelin impairs proteasome peptidase activities in podocytes.

**Figure S4** CAMP reversed the proteasome activity which was inhibited by apelin.
