# Supplementary figures and images for: Apelin promotes diabetic nephropathy by inducing podocyte dysfunction via inhibiting proteasome activities
Source: J Cell Mol Med. 2015 Jun 23;19(9):2273–85. doi: 10.1111/jcmm.12619 (PMC4568931; doi:10.1111/jcmm.12619)

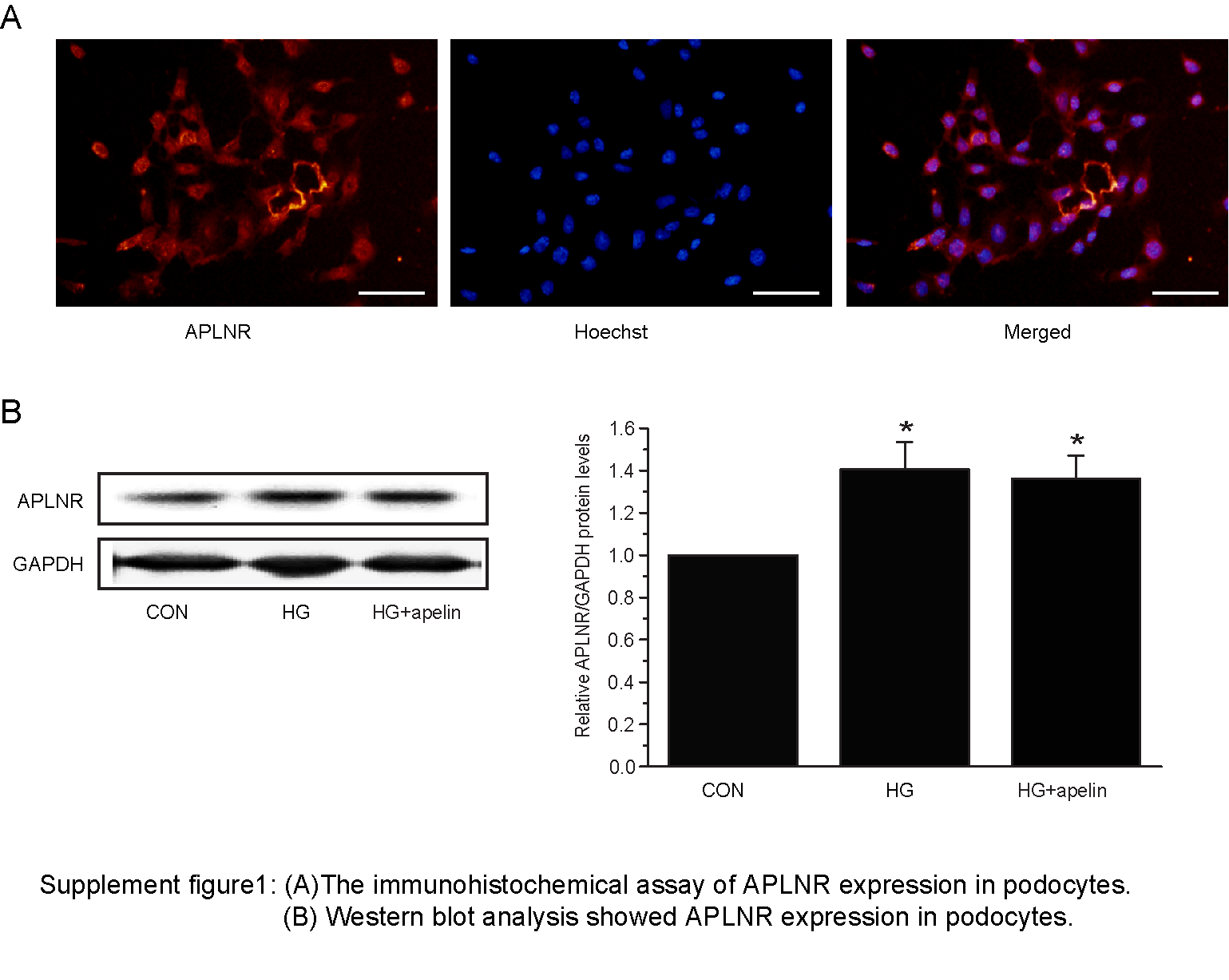

Supplement: Supplementary file 1 [file jcmm0019-2273-sd1.tif]

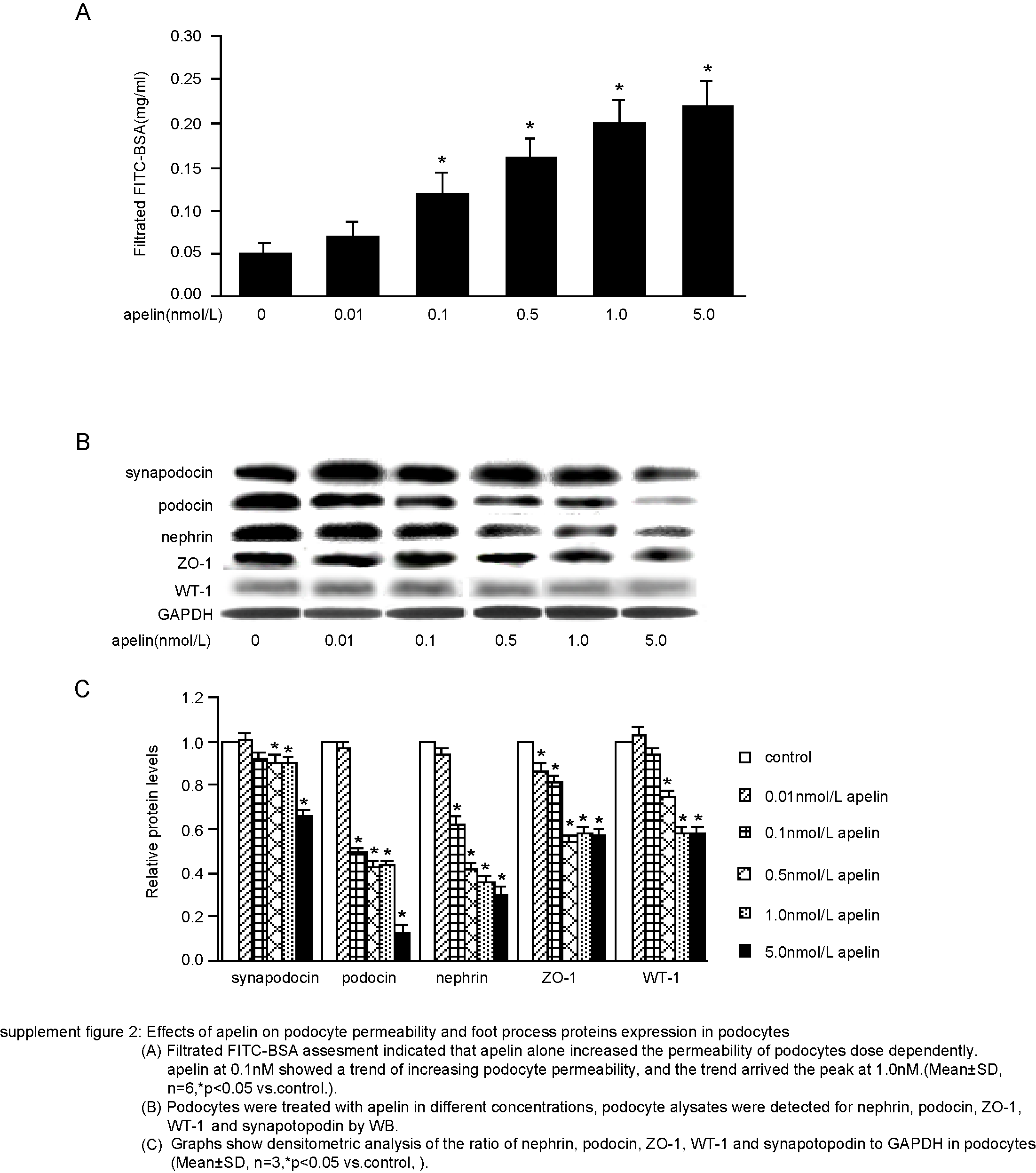

Supplement: Supplementary file 2 [file jcmm0019-2273-sd2.tif]

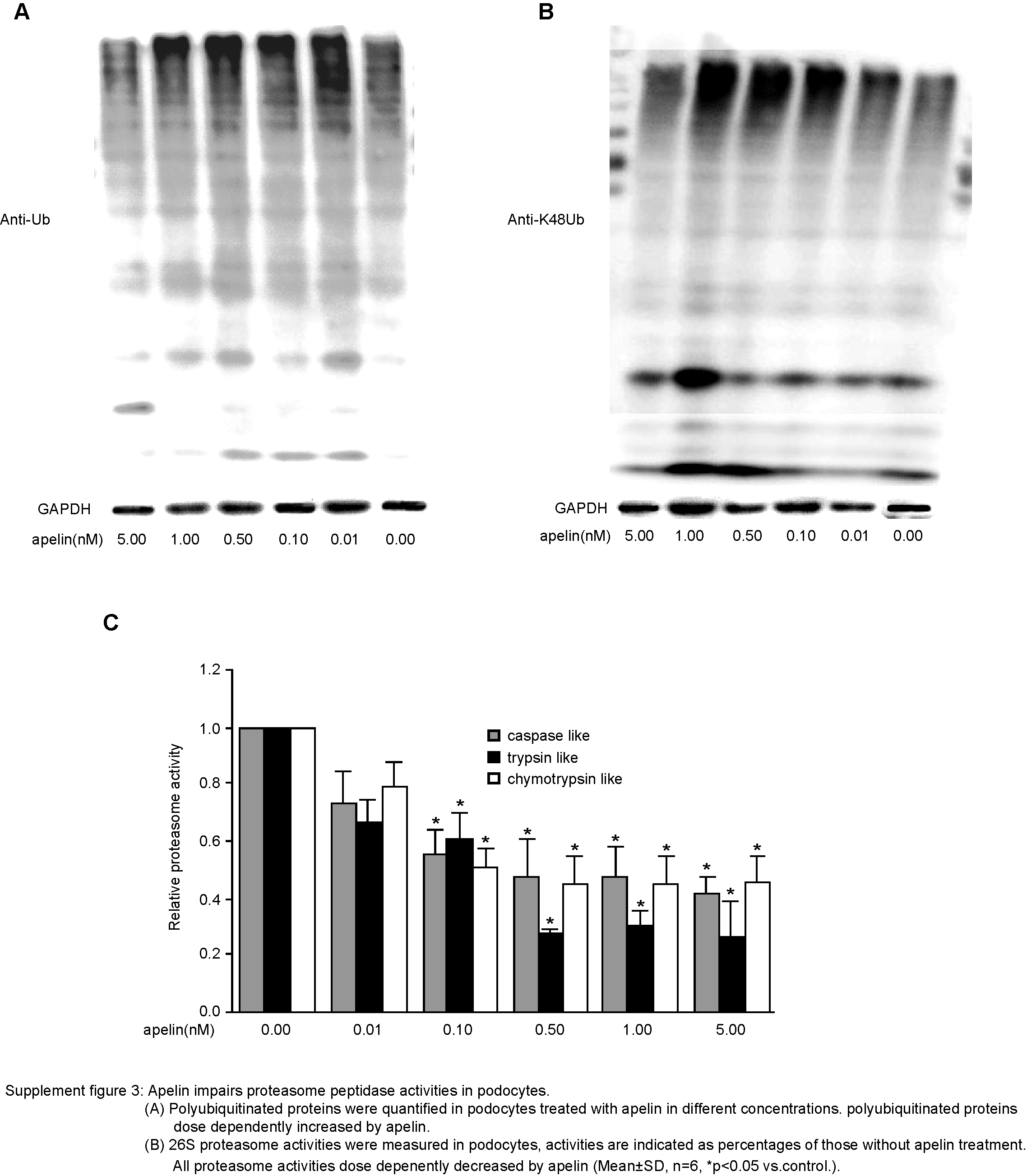

Supplement: Supplementary file 3 [file jcmm0019-2273-sd3.tif]

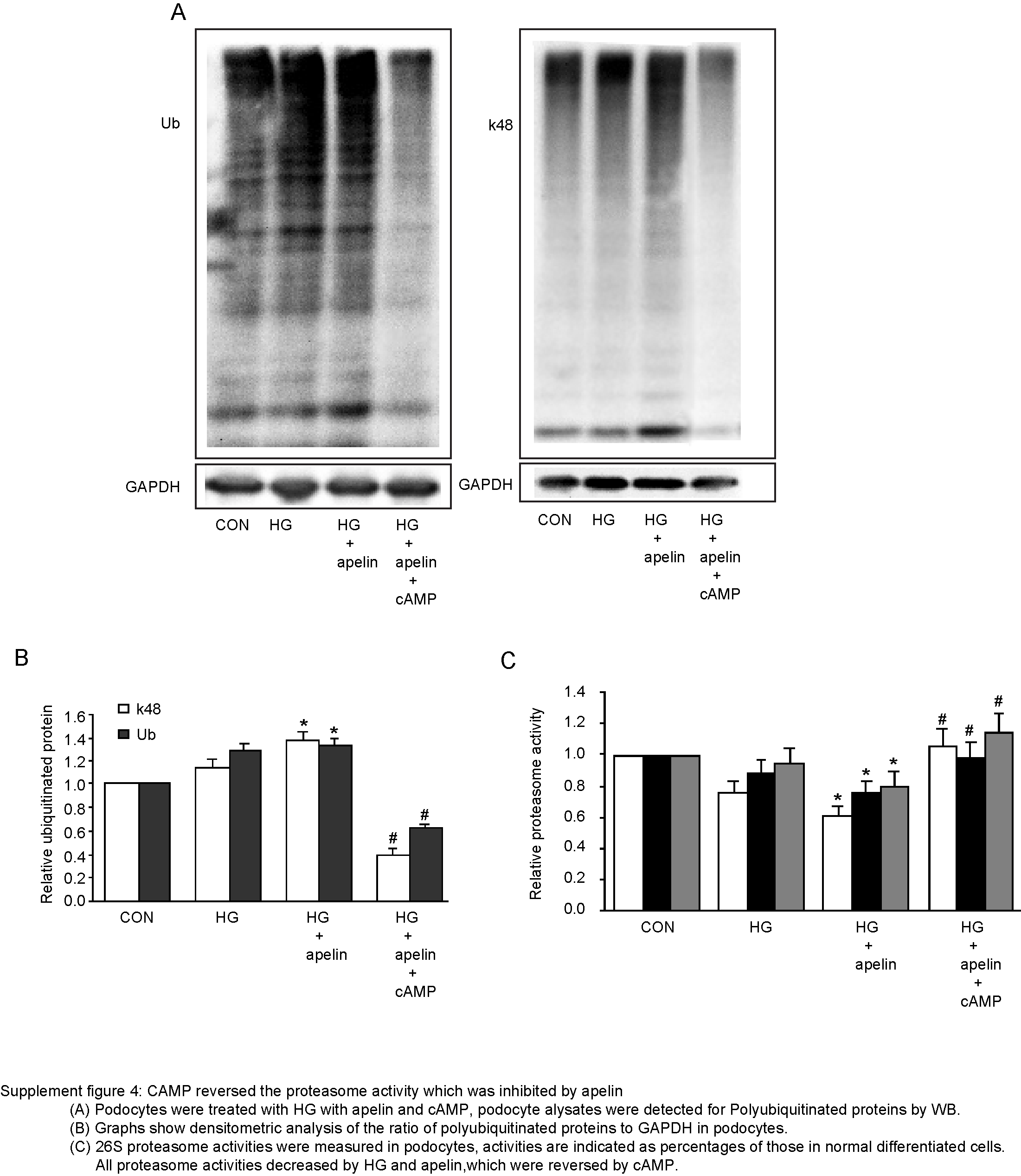

Supplement: Supplementary file 4 [file jcmm0019-2273-sd4.tif]
